# Psychological Health and Marital Adjustment in Iranian Employed Veterans and Veterans Receiving Disability Pension

**DOI:** 10.5812/ircmj.10219

**Published:** 2014-07-05

**Authors:** Fatemeh Zargar, Elham Foruzandeh, Abdollah Omidi, Abolfazl Mohammadi

**Affiliations:** 1 Department of Clinical Psychology, School of Medicine, Kashan University of Medical Sciences, Kashan, IR Iran; 2 Department of Psychology, Isfahan Science and Research Branch, Islamic Azad University, Isfahan, IR Iran; 3 Department of Psychiatry, School of Medicine, Tehran University of Medical Sciences, Tehran, IR Iran

**Keywords:** Psychological Health, Marital Status, Veterans

## Abstract

**Background:**

Human society has witnessed disasters and wars that left many consequences on families as well as social and individual life of the victims.

**Objectives:**

In this research, we compared the psychological health and marital adjustment in Iranian employed veterans with veterans receiving disability pension.

**Patients and Methods:**

The study participants were all of the veterans of Isfahan city registered in Veterans and Martyr Foundation who were receiving disability pension, were still working, or had not received any disability pension yet. A total of 330 veterans were selected by randomized systematic sampling. The Symptom Checklist-90-Revised (SCL-90-R) questionnaire and Dyadic Adjustment Scale (DAS) were completed by the participants. The data were analyzed by Chi square test, independent samples t test, and Mann-Whitney U test.

**Results:**

Almost half of the veterans did not demonstrate any psychopathology and half of them were diagnosed with borderline or serious psychopathology. Veterans receiving disability pension had more mental problems in comparison with the employed veterans. Veterans receiving disability pension had higher scores in psychosomatic disorders, obsessive-compulsive disorder, depression, anxiety, phobias, psychoticism, and total scales (general symptom index, GSI) in comparison with the employed veterans. Employed veterans and veterans receiving disability pension did not differ significantly regarding DAS scores.

**Conclusions:**

Occupational condition has an important effect on mental health of veterans.

## 1. Background

Mental health disorders are frequent among soldiers. Approximately 56% of soldiers returning from Iraq and Afghanistan have had two or more mental disorders (1). Veterans have found high rates of posttraumatic stress disorder (PTSD), depression, and related conditions (2, 3). Veterans reported high levels of anger (4-6). Studies on veterans from the World War II through the Gulf War had shown similar scores in the Minnesota Multiphasic Personality Inventory (MMPI) and MMPI-2, which means high prevalence of depression, psychosis, anxiety, paranoia, and hypochondriasis (5). About 95% of Iranian chemical and physical veterans have the global severity index (GSI) above the cutoff point in the Symptom Checklist-90-Revised (SCL-90-R) that indicates prevalent psychopathology among them. Hypochondriasis, psychosis, panic, and paranoid indexes had a higher mean score in physical and chemical veterans (7, 8). Other studies have shown that the most common psychiatric disorders among Iranian veterans and soldiers are depression (9-11), anxiety and anxiety disorders (10, 11), anger, aggression, and maladaptive behaviors (12, 13).

Studies showed that lack of suitable job is one of the most important factors affecting the psychiatric problems of veterans (14). When veterans were economically active, their complaints of depression, physical symptoms, and problems in interpersonal relationships were less (15). In these conditions, they felt happy, satisfying personal life (16), and better health (17). When a people lose their job, they loses the supports that job provides for them. This could be an important source of psychological stress (18).

Individual occupation also affects other areas of life. Research in the areas of family and employment showed transition of psychological states from one role to another (19). The soldiers with PTSD often experience job losses that affects several areas of family functioning such as family cohesion, parental satisfaction, and couple relationship (6). Soldiers who have experienced combat situations have higher level of marital dissatisfaction (20-22), relationship distress, relationship instability, sexual dysfunction (23), lower levels of cohesion and expressiveness (22), and weaker communication and intimacy (22, 23). PTSD and combat level together can also reliably predict the problems in four specific areas of marital adjustment including dyadic consensus, satisfaction, affection expression, and cohesion (24). Nearly half of the chemically injured veterans were dissatisfied with their marriage and marital relationship (24). Experiencing job loss along with other psychological, social, and family problems of veterans provides a new source for their stress. In Isfahan City, there are a significant percentage of veterans receiving disability pension (approximately 47%). Thus, study of their psychological and marital problems is necessary.

## 2. Objectives

In this research, we compared the psychological health and marital adjustment in Iranian employed veterans with veterans receiving disability pension.

## 3. Patients and Methods

This cross-sectional study was approved by Veterans and Martyr Foundation of Isfahan (No 5809/30/26220). The participants of the study were all employed veterans and veterans receiving disability pension registered by Veterans and Martyr Foundation of Isfahan up to the first half of 2010. This study was done from the second half of 2009 through the end of the year. All participants signed an informed consent form before entering the study. The purpose of study was explained to the participants. If they were unwilling to participate in the study, they were excluded. To determine the sample size, 30 employed veterans and veterans receiving disability pension were selected randomly and the study questionnaires was completed by them. Based on Cohen formula (1988) the total sample size was calculated at 261. With regard to the possible loss of subjects, the size of each group, namely, employed veterans and veterans receiving disability pension, was determined as 150. Sampling was done systematically and randomly using a list prepared by the Veterans and Martyr Foundation including veterans of more than 25% disability from three areas of the Isfahan City. A total of 300 veterans who met the inclusion criteria were recruited. The inclusion criteria were being married and having the physical ability to attend the meeting to respond to the questionnaires; the exclusion criteria were being single or divorced.

### 3.1. Measures

#### 3.1.1. The Symptom Checklist 90-Revised

SCL-90-R included 90 items to assess psychological symptoms (25). The scale has been validated in Iran (26, 27). This questionnaire measures nine scales including somatization, obsessive-compulsive disorder (OCD), interpersonal sensitivity, depression, anxiety, hostility, phobic anxiety, paranoid ideation, and psychoticism. The GSI of the questionnaire is calculated by dividing the total score on the derived 90 questions. The GSI ranges from zero to four. Scores higher than two indicate psychopathology (25). SCL-90-R is reported to have reliability indexes of more than 0.8 in most subscales in Iranian subjects (26).

#### 3.1.2. Dyadic Adjustment Scale

The purpose of Dyadic Adjustment Scale (DAS) is to assess the quality of the marital relationship or similar reciprocal relationships (28). The test consists of 32 questions in four sections: dyadic satisfaction, dyadic cohesion, dyadic consensus, and affection expression. Dyadic satisfaction shows the level of happiness in relationships and conflicts experienced by couples. Dyadic cohesion represents collaborative activities between couples. Dyadic consensus shows the couple’s agreement on the important issues such as managing finances or important decisions. Affection expression is related to how often the couple expresses their love to each other. Correlation between this measure and the Locke-Wallace Marital Adjustment test in married and divorced respondents was 0.86 and 0.88, respectively (29). The study was conducted on a sample consisting of 80 compatible and distress couples in Isfahan; the reliability coefficient of 0.59 to 0.93 for the subscales and 0.95 for the total scale was obtained (29).

Chi square test was used to determine the significance of differences between educational level and disability percent. The independent samples t-test was used to determine differences between groups regarding age, age at injury, and duration of injury. Test of normality showed that some variables had not normal distribution and Mann-Whitney U test was used to analyze them. Mann-Whitney U was performed to detect any significant difference in psychological symptoms and marital adjustment of employed veterans and veterans receiving disability pension. Evaluation of the correlation between variables such as age, age at time of injury, duration of injury, and type of injury with nine scales and total scores of SCL-90-R and DAS showed that only type of injury had significant correlation with some scales of the SCL-90-R.

## 4. Results

From 330 participants, 263 veterans filled out DAS property (110 of employed veterans and 162 of veterans receiving disability pension). In addition, 279 veterans filled out SCL-90-R property (110 of employed veterans and 169 of veterans receiving disability pension). The questionnaires with more than 25% unanswered questions were excluded from the final analysis. Table 1 shows some demographic characteristics of veterans in two groups. Table 2 provides the means, standard deviations, and comparison of SCL-90-R scales between two groups. There were significant differences between two groups in all scales except aggression, interpersonal sensitivity, and paranoia. It means that the veteran receiving disability pension had more psychosomatic complains, OCD, depression, anxiety, phobia, and psychoticism symptoms in comparison with the employed veterans. In addition they generally had more psychological problems than employed veterans. Table 3 provides the means, standard deviations, and comparison of DAS between the study groups. There were no significant differences between two groups regarding DAS subscales.

**Table 1.** Comparing the Characteristics of the Participants ^a^

Variable	Employed Veterans	Veterans Receiving Disability Pension	P value
**Age, y**	45.81 ± 5.05	45.24 ± 5.07	0.35
**Age at the Time of Injury, y**	22.22 ± 6.13	21.55 ± 5.24	0.33
**Duration of Injury**	23.97 ± 4.11	23.95 ± 3.52	0.96
**Educational Level**			0.000
Primary School	11 (9.5)	49 (28)	
Junior School	8 (6.9)	41 (23.5)	
Senior School	51 (44)	61 (35)	
≥ Bachelor	46 (39.6)	23 (13.21)	
**Disability Percent**			0.19
25-35	39 (33.6)	70 (39.5)	
36-45	29 (25)	31 (17.5)	
46-55	22 (19)	32 (18)	
56-65	14 (12)	14 (8)	
> 65	12 (10.4)	30 (17)	

^a^ Data are presented as mean ± SD or No. (%).

**Table 2.** Comparison of the Symptom Checklist 90-Revised Scales Between the Study Groups ^a^

	Employed Veterans, Mean ± SD	Veterans Receiving Disability Pension, Mean ± SD	Mann-Whitney U	Z	P value
**Somatization**	1.78 ± 0.97	2.17 ± 0.93	7159.000	-3.244	0.001
**Obsessive-Compulsive Disorder**	1.65 ± 0.89	1.95 ± 0.91	7514.000	-2.705	0.007
**Interpersonal Sensitivity**	1.52 ± 0.95	1.70 ± 0.91	8243.500	-1.597	0.110
**Depression**	1.44 ± 0.96	1.71 ± 0.98	7835.500	-2.216	0.027
**Anxiety**	1.32 ± 0.95	1.67 ± 0.97	7397.500	-2.882	0.004
**Aggression**	1.52 ± 1.01	1.85 ± 1.09	7618.000	-2.549	0.011
**Phobia**	0.98 ± 0.83	1.30 ± 0.94	7367.500	-2.931	0.003
**Paranoia**	1.52 ± 0.91	1.66 ± 0.99	8572.500	-1.098	0.272
**Psychosis**	1.16 ± 0.87	1.39 ± 0.89	7854.000	-2.189	0.029
**Global Severity Index **	1.45 ± 0.83	1.73 ± 0.83	7484.000	-2.750	0.006

^a^ Data are presented as mean ± SD.

**Table 3.** Comparison of Dyadic Adjustment Scales Between Study Groups ^a^

	Employed Veterans, Mean ± SD	Veterans Receiving Disability Pension, Mean ± SD	Mann-Whitney U	Z	P value
**Dyadic satisfaction**	36.94 ± 7.79	36.01 ± 7.77	7574.500	-1.012	0.312
**Dyadic cohesion**	13.81 ± 4.98	12.97 ± 5.35	7499.000	-1.138	0.255
**Dyadic consensus**	47.97 ± 11.07	46.15 ± 11.83	7407.000	-1.291	0.197
**Affection expression**	8.55 ± 2.69	8.19 ± 2.83	7479.500	-1.176	0.240

^a^ Data are presented as mean ± SD.

## 5. Discussion

We evaluated the mental and marital conditions of employed veterans and veterans receiving disability pension. Based on the scales of the SCL-90-R questionnaire, 47.3% of veterans had no mental disorders (total score of zero or one) and 52.7% had some kind of psychopathology (total score of three or four). The study showed that the employed veterans had a better mental status than those receiving disability pension.

This study showed that the veterans receiving disability pension had higher scores in somatization, OCD, depression, anxiety, phobia, psychoticism, and total scales (GSI) in comparison with the employed veterans. Several studies in veterans had reported similar findings (2-11). People become vulnerable to any psychiatric disorder such as psychosis (30), hypochondriasis (31), and OCD (32) by trauma, e.g. war. In general, experiencing traumatic events in late adolescence or early adulthood is associated with multiple problems in occupational, interpersonal, and social functioning (33).

In this research we found that somatization and anxiety scales had the greatest variance between SCL-90-R scales. Somatization and hypochondriasis are amongst the most common disorders in veterans (5, 8, 12). Hypochondriasis is significantly associated with physical problems, war conditions, and stressful life (31). It is shown that occupational status can lead to mental disorder. In other words, unemployment and job dissatisfaction had a significant association with depression in veterans (34). Lack of suitable job is one of the most important factors affecting the psychological status in veterans (15). On the other hand, when veterans are economically active, they have less complaints regarding depression and problems related to physical and psychological health symptoms (15, 17).

In our study, the employed veterans and the veterans receiving disability pension did not differ in subscales of DAS. Many studies showed that soldiers who experienced combat situations had problems in all the areas related to DAS (20-24, 35). Our study showed that occupational status did not significantly affect the areas related to DAS. We did not compare the veterans to nonveterans; hence, this finding does not mean that veterans had a good marital adjustment status. Our study showed that veterans in both groups did not significantly differ with regard to the level of happiness, conflict in relationships, collaborative activities, agreement on the important issues, and expressing their love. Although the veterans receiving disability pension had more psychological problems than employed veterans, the SCL-90-R subscales showed no significant difference between the veterans in both groups regarding aggression, paranoia, and interpersonal sensitivity subscales. These three subscales might have affected interpersonal relationships, especially couple relationships, more than any other subscales.

This study showed that the veterans receiving disability pension had more psychological problems than the employed ones but the marital adjustment of two groups did not differ. Most of the Iranian veterans had experienced Iraq prisons too. Although most of the Iranian veterans participated in Iran-Iraq war voluntary and studies showed less stressors in volunteer combatant prisoners than army prisoners of war (36), combat situations had affected their individual and social lives.

Based on the results of the present study, we recommend implementing these reforms by Veterans and Martyr Foundation: encourage veterans to find jobs and creating jobs adapted to their abilities; finding and recording the veteran's abilities in the past; suitable use of the existing veterans facilities, e.g. land, repair shops, bank capital, etc; lending low-rental offices to veterans; providing "the gift of employment" for veterans who received disability pension but still remain in their jobs; and assistance for part-time or hourly employment of veterans with job-state conditions.
